# Social Support and Cognitive Impairment: Results from a Portuguese 4-Year Prospective Study

**DOI:** 10.3390/ijerph18168841

**Published:** 2021-08-22

**Authors:** Ricardo Pais, Luís Ruano, Carla Moreira, Sílvia Fraga, Ofélia P. Carvalho, Henrique Barros

**Affiliations:** 1 EPIUnit-Instituto de Saúde Pública, Universidade do Porto, 4050-091 Porto, Portugal; lmruano@gmail.com (L.R.); carlamgmm@gmail.com (C.M.); silvia.fraga@ispup.up.pt (S.F.); opcarvalho20@gmail.com (O.P.C.); henrique.barros@ispup.up.pt (H.B.); 2 Departamento de Ciências da Saúde Pública e Forenses e Educação Médica, Faculdade de Medicina, Universidade do Porto, 4200-319 Porto, Portugal; 3 Unidade de Saúde Familiar Lusitana, 3514-511 Viseu, Portugal; 4 Departamento de Neurologia, Centro Hospitalar de Entre o Douro e Vouga, 4520-211 Santa Maria da Feira, Portugal

**Keywords:** social support, cognitive impairment, older people, risk

## Abstract

(1) Background: In an ageing society, social relationships may benefit cognitive performance with an impact on the health of older people. This study aims to estimate the effect of different social support sources on the risk of cognitive impairment in a sample of older Portuguese people. (2) Methods: From the Portuguese EpiPorto cohort study, we followed a sample of participants with 60 to 85 years (*N* = 656) between 2009 and 2015 (4.63 mean years of follow-up). The participants’ perception of social support from family, friends and significant others was evaluated. Cox’s regression models were used to investigate the association between this and sociodemographic variables. (3) Results: It was found that social support from friends reduces the risk of cognitive impairment. Men, participants aged 60 to 64 and those not married have a lower risk of cognitive impairment after adjusting for other variables. Participants between 80 and 85 years old (*p* = 0.021), those with less than four years of education (*p* < 0.001), and those with cognitive impairment (*p* = 0.007) have perception of less social support from friends. (4) Conclusions: A social support network from friends reduces the risk of cognitive impairment for older people.

## 1. Introduction

The increases in life expectancy we observe nowadays did not come with a proportionate increase in quality of life, as the risk of disease, disability and dementia also increases with increasing age [1]. This fact highlights the importance of quality of life in later life.

Cognitive function is an essential indicator of overall well-being in older ages. Lower scores on measures of cognitive function are associated with increased frailty and limitations to daily life activities [2,3]. Although changes in cognitive function such as recollection, familiarity, and false recognition are typical with normative cognitive ageing, cognitive decline is not a part of healthy ageing [4]. Cognitive impairment is characterised by more difficulty than expected for an individual’s age and education with memory or concentration while performing a task of everyday living or when learning new things [5]. It ranges in severity between deficits which are not clinically detected to clinically diagnosed dementia [6]. It is likely to appear prior to other disease diagnoses conducted, such as Alzheimer’s disease or dementia [7,8].

Cognitive impairment is a risk factor for dementia and mortality [9,10] as it increases dependency on others and contributes to individual vulnerability [11]. Social support can be a protective factor delaying cognitive decline among older people. Social support comprises the perception of care and assistance given within the individual social network, and it may be seen as care, financial assistance, gift-giving, counselling, or emotional assurance [12,13]. Individuals who have a variety of social relationships with family, friends, neighbours, and co-workers giving them a sense of support and affection [14] and who are involved in several social activities, such as sports and cultural activities, providing them with the sense of belonging are likely to have better health and well-being [15]. Social support is also related to better health outcomes [13,14]. Previous studies have shown that social support has a positive impact on cognition later in life and on the overall quality of life and mental health [16,17]. Insufficient social support may be a risk factor for cognitive decline, possibly due to fewer positive relationships and fewer social activities resulting in less brain stimulation and a higher risk of depression [18]. The stress-buffering hypothesis states that social support can act as a buffer against stressful life events by reducing adverse physiological stress reactions [19]. Therefore, engaging in socially and emotionally supportive environments decreases physiological reactivity and may protect against cognitive decline [20].

Research on the longitudinal impact of social support on the incidence of cognitive impairment remains unclear. A better understanding of the connection between cognitive impairment and social networks will identify areas for investing more resources and for significantly improving the quality of life at older ages [21]. Therefore, it is important to determine whether better quality marriages result in greater life satisfaction and fewer health problems, if the relationship between parents and children increases emotional support, or whether friends and neighbours are an essential source of social support for older adults [14].

The purpose of this study is to estimate the effects of different sources of social support on the risk of cognitive impairment in a population-based sample with participants over 60 years of age. It is hypothesised that higher social support from family, friends, or significant others would decrease the risks of cognitive impairment.

## 2. Materials and Methods

### 2.1. Study Population

**Figure 1:**
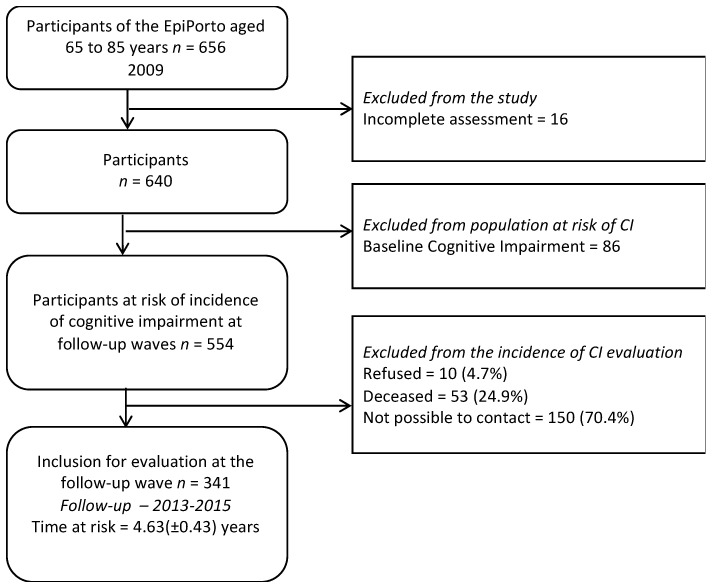
The flowchart of the study sample from 2009 to 2015. Note: “CI” refers to cognitive impairment; “Refused” refers to the participant who did not agree to participate in the follow-up surveys; “Deceased” refers to the participant who had passed away at the time of the follow-up surveys; “Not possible to contact” refers to the participants who could not be contacted for the follow-up surveys.

The present research study is based on the data from the EpiPorto cohort study. The design and methodology have been published previously [22,23]. The study protocol comprised detailed information on interviewing procedures [24]. Briefly, participants were initially contacted by letter and later by telephone in order to schedule an interview. On the appointment day, the study’s objective was explained and any concerns were clarified [25]. In 2009, 656 participants aged 60 to 85 took part in the study. Among the individuals evaluated at baseline, 16 (2.5%) were not eligible for the present study due to missing information on MMSE, and 86 (13.1%) had cognitive impairment and were excluded. The follow-up evaluation was between 2013 and 2015, and the participants were recalled for cognitive evaluation. About 213 individuals did not attend the follow-up evaluation procedure: 53 (24.9%) had died; it was impossible to contact 150 (70.4%); 10 (4.7%) refused to participate (Figure 1). There was no significant difference between the baseline data of the 341 participants and the 213 lost regarding gender or marital status. Nevertheless, participants lost in the follow-up were older (*p* < 0.001) and were less educated (*p* = 0.029) (Table 1). Among these 341 participants, 57.5% were women, 62.7% were aged between 60 and 69, most of them had 0–4 years of education (43.1%), and 70.1% were married or cohabiting.

### 2.2. Data Collection and Definition of Variables

Trained interviewers collected information on sociodemographic characteristics using structured questionnaires.

Education was recorded as completed years of schooling and further categorised into three groups: 0–4 years of education, between 5 and 9 years, and more than 10 years.

We categorised marital status into two groups: the married or cohabiting and the others (divorced, separated, widowed, or single).

We evaluated cognitive impairment using the Mini-Mental State Exam (MMSE), with cut-off points adjusted to the years of education and validated for the Portuguese population: 22 for 0–2 years; 24 for 3–6 years; and 27 for seven or more years of education [26]. Subjects would have cognitive impairment if they had an MMSE score below the age and education adjusted cut-off point.

The social support perception was assessed with the Multidimensional Scale of Perceived Social Support, which is a 12-item scale of perceived social support from family and friends. Each item scored 1 to 7, the total sum of all 12 items was a wide range from 7 to 84. The highest scores suggest high levels of social support [27].

### 2.3. Statistical Analysis

The follow-up participants were compared to losses to follow-up by using the Chi-Square test. We used the Cox proportional hazards regression models to estimate the hazard ratios (HR) and 95% confidence intervals of the association of the sociodemographic variables with cognitive impairment incidence. We used the backward stepwise conditional LR method to select the most suitable model and used Akaike’s information criterion (AIC) model selection to distinguish among the set of possible models describing the relationship between age; gender; education; marital status; social support from family, friends, or from other significant people; and cognitive impairment. The best-fit model, carrying 100% of the cumulative model weight included the variables of age, gender, marital status, and social support from friends. The Omnibus Test of Model Coefficients was statistically significant (*p* < 0.001). We complied with the model assumptions with respect to proportional risks.

We performed normality testing of social support from friends using the Skewness test; thus, we used parametric tests to compare the mean of perception of social support from friends in each variable of the study (Test-t for independent samples or One-way Anova if applicable).

The mean of social support from friends, family, and other significant people in participants with and without cognitive impairment was compared by using the General Linear Model with Bonferroni comparison, adjusted for age, sex, and marital status. Data are presented as mean and standard deviation (SD). Statistical analyses were performed with SPSS^®^ version 21 (IBM, New York, NY, USA).

## 3. Results

Three hundred forty-one participants completed the follow-up evaluation (mean follow-up of 4.63 years ± 0.43 years) of whom 297 (87.1%) maintained normal cognitive status and 44 (12.9%) had developed cognitive impairment. 

The hazard ratio of men who possessed cognitive impairment was 63% which was lower when compared to women (HR = 0.370, 95% CI = 0.184–0.744). Participants 70 to 74 years old had a hazard ratio of having cognitive impairment 229.9% higher than participants who were aged 60 to 64 years old. Furthermore, participants 75 to 79 years old had a hazard ratio of 212.8% higher. The hazard ratio for the divorced or separated and the widowed or the single for having cognitive impairments was 60.2% lower when compared to married participants (HR = 0.398, 95% CI = 0.186–0.852). The increase in social support from friends reduces the hazard ratio of cognitive impairment by 23% (Table 2).

No significant differences for the perception of social support from friends were observed concerning gender or marital status, except for age and education. Participants more than 80 years old had a lower perception of social support from friends than participants with 60 to 64 (mean = 4.087; SD = 1.288 vs. mean= 4.882; SD = 1.625, *p* = 0.021), and participants with fewer years of education had a lower perception of social support from friends (mean = 4.450, SD = 1.544 vs. mean = 5.257, SD = 1.167; *p* < 0.001) (Table 3).

A lower perception of social support is associated with cognitive impairment (mean = 5.038, SD = 0.624; *p* = 0.007), specifically social support from friends (mean = 4.413, SD = 0.885; *p* = 0.015) or social support from significant others (mean = 5.517, SD = 0.657; *p* = 0.017) adjusted for sex, age, and marital status (Table 4).

## 4. Discussion

This study investigated the impact of social support on the incidence of cognitive impairment using a representative population-based sample of Portuguese older people during 4.6 mean years of follow-up. It was concluded that social support from friends decreases the hazard ratio of cognitive impairment.

With increasing age, older people have fewer social interactions, and most of the social interactions occur with family members. At the onset of this study, we expected that social support from family would have an impact on cognitive impairment. We also expected that married people would be less at risk of cognitive impairment than divorced, separated, widowers, or single individuals. Some studies report that being married when compared to being a widower has a protective effect against cognitive impairment [16,28]. However, the results from this study do not support those initial expectations as a statistically significant relationship between social support from family and cognitive impairment has not been found. In fact, the group composed of divorced, separated, widowed, and single participants had a lower hazard ratio for cognitive impairment than the one consisting of married participants. Murata et al. (2017) reports that support from family may be an obligation and may sometimes be misunderstood, whereas support from friends is voluntary and often involves activities of common interest mostly outside of the home, which arguably may provide increased physical and cognitive stimulation [13].

Some studies value the importance of social support in the cognitive function of older people [12]. Weng et al. (2020) posits that positive relationships and more social activities results in more brain activity and less depression [18]. Brown et al. (2009) studying a neighbourhood context claims that support from friends has more impact on cognitive function than support from family [29], whereas Noguchi et al. (2019) adds that friends are typically of the same age, share the same experiences, and have similar lifestyle and geographical proximity, which also acts on reducing loneliness [12]. All these lines of evidence support the main finding of this study, which suggests that social support from friends decreases the hazard ratio of cognitive impairment.

We did not find differences in either gender or marital status in the perception of social support from friends, but we did find differences regarding age and years of education. Older and less educated participants have a lower perception of social support. For older participants, this perception may be reflecting the decrease in social interactions observed as age increases [16]. Smith et al. (2018) reported that social isolation was more frequent in less educated participants [30] and postulated that this may be due to a reduced social network membership.

The lower perception of social support is associated with cognitive impairment even after adjusting for sex, age, and marital status. This agrees well with other studies [12,13,29], which also find that people with cognitive impairment have less participation in the community and fewer interactions and access to social resources [21].

The observed protective effect of social relations could be due to reverse causality, being the cause of less social interactions rather than the consequence of it. We tried to control for this effect by excluding participants who had cognitive impairment at the baseline.

In agreement with other studies, men are at lower risk of cognitive impairment [31,32], with Laws et al. citing hormonal differences as a cause due to a lack of oestrogen in women [33].

Ageing is consistently associated with an increased risk of cognitive impairment [34,35,36], as we also report.

The major strength of our study is the prospective study design and the exclusion of participants with cognitive impairment at baseline.

There are some limitations to the present study. The first is assessing cognitive function by using the MMSE scale without clinical assessment or any other tests. Despite, MMSE being the most cited small-sized scale used for dementia and cognitive impairment assessment and despite being considered a reliable and valid test for cognitive impairment [25,37], clinical assessment or validation by using other tests would have provided further confirmation. Similarly, the use of the MSPSS scale to assess social support, which is targeted to assess the perception of social support but not the social support actually received, is another limitation. Secondly, we could not distinguish the relatives who provided social support, for example, if they were the spouse, children, or other family members. The participants lost to follow-up were older and had fewer years of education than the participants included in our study and, therefore, had a higher risk of cognitive impairment. This could have resulted in an underestimation of the new cases of cognitive impairment. The inability to diagnose dementia meant that we could not exclude participants with dementia from the study and, therefore, may have overestimated some of the results.

## 5. Conclusions

In conclusion, this prospective study allows us to confirm the importance of social support from friends in reducing the risk of cognitive impairment. Participants aged 80–85 years old or with fewer years of school had a lower perception of social support. Recognising the impact of social support, especially social support received from friends, can be useful for health professionals to improve their care provision and better advise their users. It can also contribute to the definition of health promotion policies that favour social networks through the development of supporting community groups, neighbourhood help groups, or social support services for older people.

## Figures and Tables

**Table 1:** Sociodemographic characteristics of participants.

Characteristic	Follow-Up	Lost to Follow-Up	*p*-Value
*N*	341	213	
Gender			
Female	196 (57.5)	133 (62.4)	0.247
Male	145 (42.5)	80 (37.6)	
Age (Years)			
60–64	114 (33.4)	30 (14.1)	<0.001
65–69	100 (29.3)	40 (18.8)	
70–74	71 (20.8)	44 (38.3)	
75–79	36 (10.6)	55 (25.8)	
80–85	20 (5.9)	44 (20.7)	
Education			
0–4	147 (43.1)	116 (54.7)	0.029
5–9	82 (24.0)	40 (18.9)	
≥10	112 (32.8)	56 (26.4)	
Marital Status			
Married/Cohabiting	239 (70.1)	133 (62.4)	0.062
Divorced, Separated, Widower, Single	102 (29.9)	80 (37.6)	

Note: Data are *n* (%); *p*-value compares follow-up to lost to follow up, obtained with Chi-square test.

**Table 2:** The multivariable Cox analysis of gender, age, marital status, and social support on cognitive impairment.

Characteristics	HR (95% CI)
Gender	
Female	Reference
Male	0.370 (0.184–0.744)
Age	
60–64	Reference
65–69	0.857 (0.321–2287)
70–74	3.299 (1.383–7.868)
75–79	3.128 (1.097–8.922)
80–85	1.013 (0.205–5.005)
Social Support	
Friends	0.770 (0.635–0.933)
Marital Status	
Married/Cohabiting	Reference
Divorced, Separated, Widower, Single	0.398 (0.186–0.852)

Note: HR, hazard ratio; 95% CI, 95% confidence interval.

**Table 3:** Perception of social support from friends mean (±SD) according to sociodemographic variables.

Characteristics	Social Support Perception	*p*-Value
Gender		
Female	4.805 (1.476)	0.694 (a)
Male	4.866 (1.342)	
Age		
60–64	4.882 (1.288)	0.021 (b)
65–69	4.790 (1.454)	
70–74	4.736 (1.552)	
75–79	5.382 (1.155)	
80–85	4.087 (1.625)	
Education		
0–4	4.450 (1.544)	<0.001 (b)
5–9	4.927 (1.333)	
≥10	5.257 (1.167)	
Marital Status		
Married/Cohabiting	4.747 (1.395)	0.095 (a)
Divorced, Separated, Widower, Single	5.027 (1.463)	

Note: Data are means (±SD); *p*-value compares mean between groups, obtained with (a) Independent samples *t*-test; (b) One-way ANOVA test.

**Table 4:** Perception of social support mean (±SD) according to cognitive status.

Social Support	NCI	CI	*p*-Value ^a^
Family	5.529 (0.294)	5.180 (0.734)	0.071
Friends	4.979 (0.353)	4.413 (0.885)	0.015
Other Significant	5.593 (0.262)	5.517 (0.657)	0.017
Total	5.483 (0.25)	5.038 (0.624)	0.007

Note: ^a^ adjusted for sex, age, and marital status; NCI: no cognitive impairment; CI: cognitive impairment; *p*-value obtained with the General Linear Model with Bonferroni comparison; data are means (±SD).

## Data Availability

Restrictions apply to the availability of these data. Data were obtained from [38] and are available (https://ispup.up.pt) (accessed on 20 April 2021) with the permission of [38].
